# Lipomatous metaplasia identified in rabbits with reperfused myocardial infarction by 3.0 T magnetic resonance imaging and histopathology

**DOI:** 10.1186/1471-2342-13-18

**Published:** 2013-07-01

**Authors:** Yuanbo Feng, Feng Chen, Yi Xie, Huaijun Wang, Marlein Miranda Cona, Jie Yu, Junjie Li, Jan Bogaert, Stefan Janssens, Raymond Oyen, Yicheng Ni

**Affiliations:** 1 Radiology Section, Department of Imaging and Pathology, University Hospitals, KU Leuven, Belgium; 2 Molecular Small Animal Imaging Center (MoSAIC), Biomedical Sciences Group, University Hospitals, KU Leuven, Belgium; 3 Departments of Electronics and Information System (ELIS), Ghent University, Ghent, Belgium; 4 Department of Cardiovascular Diseases, University Hospitals, KU, Leuven, Belgium

**Keywords:** Acute myocardial infarction, Animal model, Chronic ischemia, Cardiovascular magnetic resonance imaging, Lipomatous metaplasia

## Abstract

**Background:**

Cardiac lipomatous metaplasia (LM) occurs in patients with chronic ischemic heart disease and heart failure with unclear mechanisms. We studied coronary occlusion/reperfusion-induced myocardial infarction (MI) in rabbits during a 9-months follow-up using 3.0 T magnetic resonance scanner, and confirmed the presence of MI in acute phase and LM in chronic phase using histopathology.

**Methods:**

MI was surgically induced in 10 rabbits by 90-min coronary artery occlusion and reperfusion. Forty-eight hours later, multiparametric cardiac magnetic resonance imaging (cMRI) was performed at a 3.0 T clinical scanner for MI diagnosis and cardiac function analysis. Afterwards, seven rabbits were scarified for histochemical staining with triphenyltetrazolium chloride (TTC), and hematoxylin-eosin (HE), and 3 were scanned with cMRI at 2 days, 2 weeks, 2 months and 9 months for longitudinal observations of morphological and functional changes, and the fate of the animals. Post-mortem TTC, HE and Masson's trichrome (MTC) were studied for chronic stage of MI.

**Results:**

The size of acute MI correlated well between cMRI and TTC staining (r^2^=0.83). Global cardiac morphology-function analysis showed significant correlation between increasing acute MI size and decreasing ejection fraction (p<0.001). During 9 months, cMRI documented evolving morphological and functional changes from acute MI to chronic scar transformation and fat deposition with a definite diagnosis of LM established by histopathology.

**Conclusions:**

Acute MI and chronic LM were induced in rabbits and monitored with 3.0 T MRI. Studies on this platform may help investigate the mechanisms and therapeutic interventions for LM.

## Background

The moderate size rabbit model of occlusion/reperfusion-induced myocardial infarction (MI) has been used in cardiac imaging research [1-3]. In particular, its versatile advantages have been demonstrated in cardiac magnetic resonance imaging (cMRI) studies at a 1.5 T clinic magnet [2]. 3.0 T MRI scanner with 8-channels or more cardiac array coils can obtain cardiac images with higher signal-to-noise-ratio, temporal and spatial resolutions, and shorter acquisition time compared to the conventional 1.5 MRI scanner [4,5]. However, there are few reports on the evaluation of rabbit model of occlusion/reperfusion-induced MI by 3.0 T scanner. In this experiment, we applied a 3.0 T magnet to re-evaluate the rabbit model on both the acute and chronic phases of MI.

Myocardial fat develops with aging and is commonly seen at right ventricular (RV) free wall and RV outflow tract in humans. Substitution of myocardial tissue by fat is less common in the left ventricle, except for some pathologic conditions such as healed MI, dysplasia, cardiac lipoma, tuberous sclerosis complex, dilated and dystrophic cardiomyopathy, etc. [6]. Myofibroblasts are supposed to play an important role in myocardial healing after MI. These cells produce collagen to constitute scar tissue, prevent infarct expansion, and stabilize ventricular wall of the heart [7]. Cardiac lipomatous metaplasia (LM) refers to the adipose tissue present in the cardiac wall to replace scar tissue within an infarcted territory. Recently, one clinical report showed that the LM occurred more often in hyperlipoproteinaemia patients and in 11% patients with chronic ischemic heart disease (CIHD) [8]. Another paper indicates that 68% of scars associated with CIHD showed the LM on autopsy [9]. How the collagen fiber degenerates into adipose tissue is still unclear. Findings of LM have been seldom described in veterinary subjects [10], yet never established by both *in vivo* and *ex vivo* animal research.

The purposes of this study were as follows: a) longitudinal evaluation of MI by a 3.0 T MRI scanner, and b) comparison of MI between MRI and histopathology findings on both acute and chronic phase. This way, we incidentally identified the presence of LM in rabbits. In this report, we describe such findings and further discuss their implications for translational cardiologic research by cross-referencing pertinent literature.

## Methods

### Animal models

**Figure 1:**
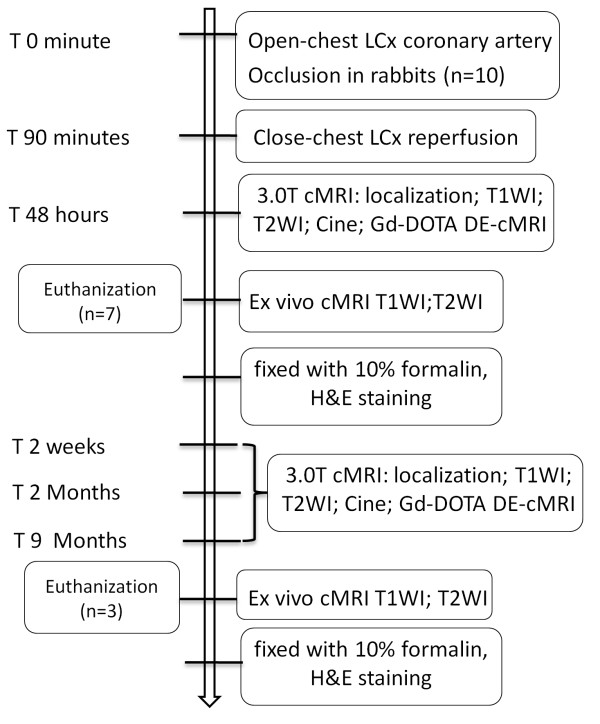
**Flowchart of experimental procedure.** LCx: left circumflex; cMR: cardiac magnetic resonance; T1WI: T1 weighted imaging; T2WI: T2 weighted imaging; DE: delayed enhancement; HE: hematoxylin and eosin.

This study was approved by the institutional ethical committee for animal care and use. Ten male New Zealand white rabbits (Animal House, K.U. Leuven, Belgium) weighing 3.0 kg were sedated, endotracheally intubated and mechanically ventilated. The rabbit received intravenous (i.v.) injection of pentobarbital (Nembutal; Sanofi Sante Animale, Brussels, Belgium) at 40 mg/kg/h to maintain anesthesia during the open-chest operation. A left-side thoracotomy was performed at the fourth intercostal space and a suture was placed underneath the left coronary artery. Reperfused MI was induced by tying the suture with a single detachable knot. Ninety minutes after coronary occlusion and sixty minutes after closure of thoracic cavity, the knot was detached outside the thorax by pulling the exteriorized end of suture in the closed-chest condition [2]. As shown in the flowchart of the experimental procedure (Figure 1), all the animals were imaged for acute phase evaluation with cMRI, 3 rabbits were randomly served as chronic MI models and underwent cMRI 48 hours, 2 weeks, 2 months and 9 months after acute MI for longitudinal observations on morphological and functional changes as well as the fate of the animals.

### cMRI

The rabbit was gas-anesthetized with 2% isoflurane in the mixture of 20% oxygen and 80% room air, through a mask connected via a tube to a ventilation instrument (Holliston, MA, USA) and placed supinely in a holder. Using a commercial 8-channal phased array knee coil, cMRI was performed at a 3.0 T clinical MRI scanner (Trio, Siemens, Erlangen, Germany) with a maximum gradient capability of 45 mT/m. The acquisition of cMRI was triggered by ECG and gated by the respiration using a small animal monitoring and gating system (SA Instruments, Inc. Stony Brook, New York). The two surface ECG electrodes were attached to the shaved thorax skin of apparent apical pulse and the left leg. The respiration control sensor was attached on the middle of the abdomen. As shown in Table 1, cardiac morphology and edema were inspected with T1 weighted imaging (T1WI) and T2 weighted imaging (T2WI), respectively. The cine-MR images were acquired in the short-axis, vertical long-axis and horizontal long-axis planes for displaying cardiac contraction. Each cine-MRI consisted of 25 frames, spaced equally across the cardiac cycle, with the acquisition time of 2.5 min. To evaluate MI, 3D delayed-enhancement (DE-cMRI) was acquired 20 min after bolus iv injection of Gd-DOTA (Dotarem, Guerbet, France) at 0.1 mmol/kg. The ex vivo cMRI on rabbit heart was described previously [2].

**Table 1:** Parameters of cMRI sequences with a 3.0 T clinic scanner without respiration holding

	**T1WI In vivo**	**T2WI In vivo**	**Cine-MRI In vivo**	**DE-MRI In vivo**	**T1WI Ex vivo**	**T2WI Ex vivo**
**Sequence type**	TSE	TSE	True-FISP	IR-turbo-FLASH	SE	SE
**Repetition time (ms)**	621	750	357	396	2300	3200
**echo time (ms)**	15	74	1.6	1.54	4.12	490
**Field of view (mm**^**2**^**)**	240×195	240×195	240×195	240×180	512×464	512×464
**Filp angle (°)**	180	180	60	15	9	0
**Bandwidth (Hz/Px)**	305	235	751	300	350	434
**In-plane resolution (mm**^**2**^**)**	0.9×0.9	0.9×0.9	1.2×0.9	1.1×0.8	0.5×0.5	0.6×0.6
**Slice thickness (mm)**	3.0	3.0	3.0	3.0	0.5	0.6
**Slices**	8	8	1	10 (3D)	64 (3D)	52 (3D)
**Number of averages**	6	6	3	1	1	4
**Inversion time (ms)**	(−)	(−)	(−)	360	900	(−)
**Fat suppress**	No	No	Yes	Yes	Yes(Figure 6)	Yes
					No(Figure 7)	
**Total acquisition time**	15 min 5 s	7 min 32 s	2 min 50s	1 min	5 min 21 s	10 min 10 s
**Phase**			25 frames/cycle			
**Contrast agent (mmol/kg)**				0.1		

*TSE* Tubo Spin echo, *True-FISP*: true fast imaging with steady-state precession; *IR-turbo-FLASH*: A segmented k-space inversion recovery turbo fast low angle shot.

### Postmortem histochemical staining

After the cMRI scans at day 2 and month 9, the rabbits were euthanized with overdosed pentobarbital. The excised heart was imbedded in and filled with 3% agar solution of 40°C in a Plexiglas heart matrix for ex vivo cMRI after cooling at −20°C for 15 min. After MRI, the imbedded heart was cut into 3 mm short axis slices similar to that with *in vivo* cMRI. The slices were incubated at 37°C in 2% triphenyltetrazolium chloride (TTC) solution for 15 min and fixed with 10% formalin for 24 hours. The heart sections in the fresh condition, shortly after TTC staining, and 24 hours after formalin fixation were photographed with a digital camera. One day later, heart sections were imbedded into paraffin-blocks, which were then cut into 5 micron thick slices and stained with Masson's trichrome (MTC) and hematoxylin -eosin (HE) for microscopic assessment. MTC staining is used for the detection of collagen fibers in the heart, where the collagen fibers are stained blue and the nuclei are stained black and the background myocardium is stained red. HE and MTC macro- and microscopic photographs were further co-localized and interpreted cross-referencing cMRI findings.

### Data analysis

The global LV parameters including end-diastolic volume (EDV), end-systolic volume (ESV), stroke volume (SV), ejection fraction (EF), cardiac output (CO), and LV-mass were determined by tracing the endocardial and epicardial contours on end-distolic and end-systolic cine images with a commercially available software Syngo Argus (Siemens, Germany). The papillary muscles were included in LV mass measurement. Mass in grams was calculated assuming a specific gravity of 1.05 g/cm3.

The planimetry of the percent of MI size on DE-cMRI and postmortem digital photographs were estimated on built in software of the system (SyngoMR A30) and the software Image J 1.38x (Research Services Branch, NIH, Bethesda, MD, USA). All the images were in different order, the estimation of MI size on cMRI and on pathology was performed by two authors separately.

The MI size per slice by MRI was assessed semiautomatically by computer counting of all enhanced pixels within the short-axis image. Enhanced pixels were defined as pixels with signal intensities more than two standard deviations above the mean of image intensities in the remote normal myocardial region on the same images [11]. The sum of enhanced pixels from each of the 6 to 8 short-axis images divided by the total number of pixels within the LV myocardium multiplied by 100%. The global MI size was determined as a percent of left ventricle mass volume (%LV).

The signal intensity (SI) ratio refers to myocardial lesion comparable to normal myocardium on T1WI and T2WI, which was performed on the Siemens workstation using the built-in software by two authors with consensus. For the quantification of SI ratios, a round of region of interesting about 1 cm2 was drawn on the lesion and normal myocardium.

### Statistical analyses

Data were reported as mean ± standard deviation (SD). Statistical analysis was performed with Graphpad Prism 5 software. The correlation relationship between EF and MIS was compared by the linear correlation analysis. The agreement between DE-cMRI and TTC measurements of infarct size was assessed by linear correlation analysis. A difference was considered statistically significant if the P value was less than 0.05.

## Results

### General conditions

All rabbits tolerated well the procedures including systemic anesthesia, open-chest surgery, and cMRI. Seven rabbits were used for acute phase and 3 for chronic phase of MI studies. The presence of MI was evident by typical ECG alterations initially, on cMRI during follow-up and with histomorphology at the end. The 3 rabbits gained body weight from 3.0 to 4.2 kg during the 9 months after the MI insult. They manifested normal behaviors and diet without symptoms of heart failure such as dyspnea, tachycardia, tachypnea, cyanosis, stupor, arrhythmia, and hypothermia. There was no apparent difference that allowed distinguishing one from the other rabbits.

### cMRI and histopathology findings 48 hours after acute MI

**Figure 2:**
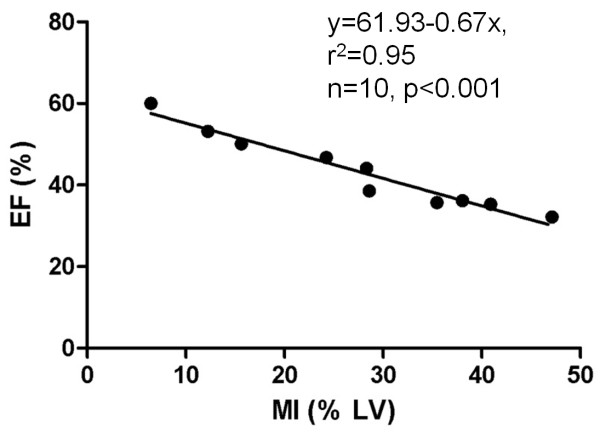
**Cardiac global morphology-function analysis.** In vivo cMRI enabled to establish the relationship between MI (hyperenhanced region on DE-cMRI) and EF (global LV function measured on cine-MRI) in rabbits with reperfused MI at 48 hours. The increasing MI size correlates significantly with the decreasing EF (p<0.001), confirming a successful model of acute reperfused MI.

Cardiac global morphology-function analysis on cine-MRI and DE-cMRI showed that the increasing MI size significantly correlates with the decreasing EF (r^2^=0.95; p<0.001), confirming a successful model of AMI (Figure 2).

**Figure 3:**
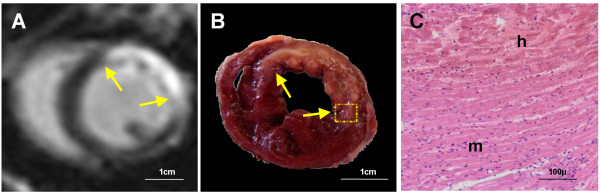
**A short axis midventricular section shown on cMRI and histopathology in a rabbit with 48-hours reperfused MI. A**: DE-MRI shows a hyperenhanced transmural zone at the anterior lateral wall with sporadic hypointense spots corresponding to intramural hemorrhage. **B**: The pale region with dark red spots on TTC histochemically stained section corresponds to hyperenhanced region with hypointense spots on DE-MRI. Dashed square indicates where microscopy was focused. **C**: microscopic view (magnification×100) of HE stained slide proves the presence of reperfused hemorrhagic acute MI. h: hemorrhagic infarction; m: adjacent myocardium with inflammatory infiltration.

**Figure 4:**
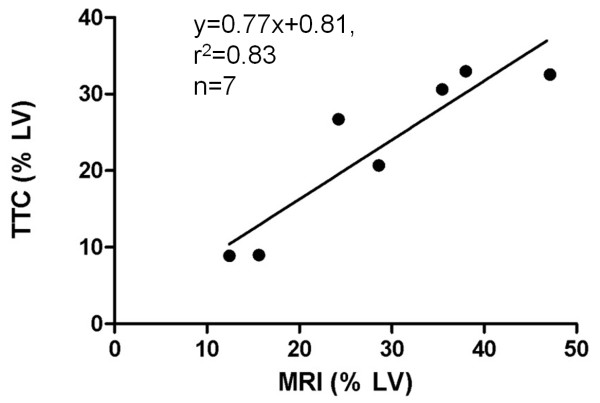
**Comparing the global MI size between in vivo cMRI and TTC histochemical staining in rabbits with reperfused MI at 48 hours.** The linear regression correlation test shows the high correspondence on MI (% LV) between MRI and TTC.

The average sizes of MI (% LV) on DE-cMRI and TTC were 35.41% ± 12.2% and 31.62%± 13.74%, respectively with no significant difference between them (p=0.8). The representative MI on DE-MRI and histopathology of acute MI were shown in Figure 3. A hyperenhanced transmural zone with sporadic hypointense spots at the anterior lateral wall on DE-MRI (Figure 3A). The pale region with dark red spots on TTC histochemically stained section (Figure 3B) corresponded to hyperenhanced region with hypointense spots on DE-MRI (Figure 3A). Microscopic view of HE stained slide proved the presence of acute reperfused hemorrhagic MI (Figure 3C). The linear regression correlation test showed the high correspondence (r^2^=0.83) on MRI and TTC (Figure 4).

### Longitudinal cMRI findings during 9 months

The global functional parameters of EDV, ESV, SV, CO, EF and LV-mass were derived from cine cMRI for 48 hours and 9 months after MI and listed in Table 2. Although the small sample size did not allow comprehensive statistical analyses, a considerable increase of the EF was noticed in these 3 rabbits after 9 months of MI.

**Table 2:** Quantitative parameters of global LV function on 48 hours and 9 months

	**48 Hours**	**9 Months**
**Parameters**	**Mean ± SD**	**Mean ± SD**
HR (bpm)	134±16	123±15
EDV (ml)	4.36±0.94	4.71±1.04
ESV (ml)	2.97±0.77	2.75±0.57
SV (ml)	1.35±0.11	1.95±0.38
CO (l/min)	0.18±0.04	0.24±0.08
EF (%)	29.63±2.73	41.69±1.93
Myoc.m(g)	3.31±0.38	2.71±0.72

*HR* heart rate, *EDV* end diastolic volume, *ESV* end systolic volume, *SV* stroke volume, *CO* cardiac output, *EF* ejection fraction, *Myoc.m* myocardial mass.

HR acquired under anesthesia.

**Figure 5:**
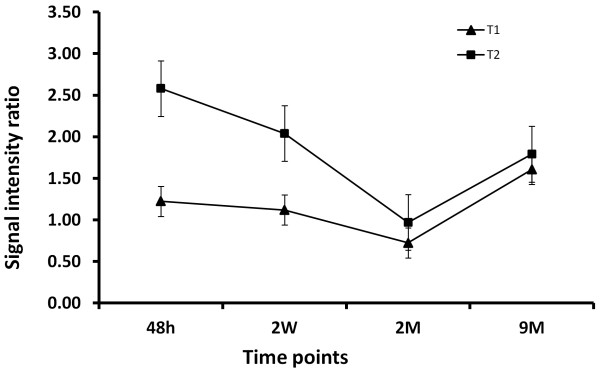
**The changes of signal intensity (SI) ratio with time on T1WI and T2WI of three LM cases.** On T1WI, the SI ratio remained almost the same on 48 hours and 2 weeks after MI, decreased 2 months later indicating the formation of fibrotic scar; but elevated drastically after 9 months suggesting the substitution of fibrosis with the LM. On T2WI, the high SI ratio declined gradually from 48 hours to 2 weeks reflecting the serious to resolved edema, followed by a normalized ratio on 2 months due to fibrosis. The ratio after 9 months increased again due to the LM. The ratio changes on both T1WI and T2WI display a step-by-step evolution from myocardial necrosis through fibrosis to adipose substitution.

The longitudinal changes of SI ratio on T1WI and T2WI averaged from three LM animals were showed in Figure 5. On T1WI, the SI ratio was closed to 1 from 48 hours and 2 weeks after MI, the ratio dropped below 1 in 2 months, but rebounded to about 1.5 after 9 months. On T2WI, the SI ratios were about 2.5 and 2 on 48 hours and 2 weeks due to the serious edema during the first two weeks, decreased to about 1 on 2 months as a result of fibrosis; and increased to 1.5 after 9 months with the LM formation. The ratio changes on both T1WI and T2WI display a step-by-step evolution from myocardial necrosis through fibrosis to adipose substitution.

**Figure 6:**
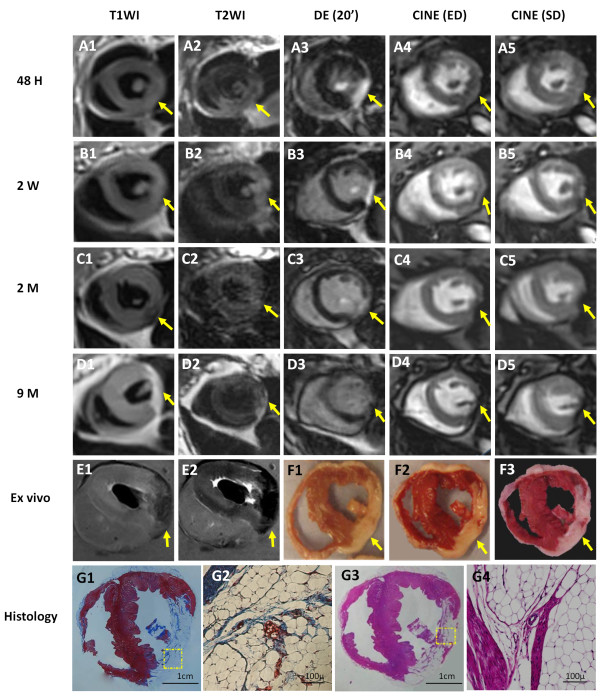
**Longitudinal evaluation of progressive changes from infarcted myocardium to large LM (arrows) by in vivo, ex vivo cMRI and histological study.** Serial images of the midcardiac slice by T1WI (A1-D1), T2WI (A2-D2), DE-MRI (A3-D3) and Cine-MRI (A4-D4 and A5-D5) were taken at 48 hours (A1-A5), 2 weeks (B1-B5), 2 months (C1-C5) and 9 months (D1-D5), respectively. Acute (48 hours) MI (A1-A5): The infarct appeared isointense on T1WI (A1); hyperintense including papillary muscle on T2WI (A2); hyperenhanced on DE-cMRI (A3); and isointense but hypokinetic on Cine-MRI (A4, A5). Early (2 weeks) chronic MI (B1-B5): The infarct remained isointense on T1WI (B1); hyperintense but smaller on T2WI (B2); hyperenhanced transmural on DE-MRI, thinner than before (B3); and dyskinesia of the lateral wall on Cine-MRI (B4, B5). Chronic (2 months) MI (C1-C5): slightly hypointense on T1WI (C1); almost isointense with irregular, boundary on T2WI (C2); moderately enhanced on DE-MRI (C3); partially signal lost on the fat suppression Cine-MRI, suggesting the initiation of LM (C4, C5). Healed (9 months) MI (D1-D5): hyperintense with clear boundary on T1WI (D1); moderately hyperintense on T2WI (D2); lack of contrast enhancement on DE-MRI (D3); and signal void on cine-MRI with fat suppression (D4, D5). Postmortem T1WI and T2WI MRI with fat suppression (E1, E2): drastic signal losses corresponded to the high signal on in vivo T1WI and T2WI (D1, D2), suggesting large LM. Macroscopies (F1: fresh section; F2: 15 min after TTC staining; F3: 24 hours after TTC staining) corresponded well with MR images. Fat extensively occupied TTC-negative region. Histology (G1-G4): MTC stained photograph (G1) and photomicrograph (G2) (magnification, ×100) and H&E stained photograph (G3) and photomicrograph (G4) (magnification, ×100) demonstrated clearly LM, with high densities of the adipose cells surrounded by few fibers and macrophages. Dashed square indicates where microscopy was focused.

The gradual identification of LM formation was shown in Figure 6.

Acute MI stage (Figure 6 A1-A5): 48 hours after reperfused MI, the infarcted region appeared almost isointense on T1WI, but hyperintense to a larger extent at the lateral and inferior wall involving a papillary muscle (LCx supplied area) on T2WI. DE-cMRI revealed a hyperenhanced area at the lateral and inferior wall, which was smaller than that shown on T2WI (Figure 6 A2). Cine-cMR showed homogenous signal but hypokinetic on the lateral wall (Figure 6 A4, A5). These were similar to what have been described for rabbits with acute MI [10].

Early chronic MI (Figure 6 B1-B5): 2 weeks after MI, the infarct remained isointense on T1WI and hyperintense on T2WI, but the hyperintense area on T2WI became smaller than that at 48 hours (Figure 6 B2 vs. A2), suggesting resolved edema. DE-cMRI showed hyperenhancement both at the lateral wall and papillary muscle (Figure 6 B3), which was thinner than that seen at 48 hours (Figure 6 A3). Cine-cMRI confirmed dyskinesia of the lateral wall (Figure 6 B4, B5).

Chronic MI (Figure 6 C1-C5): 2 months after MI, the lesion became slightly hypointense on T1WI (Figure 6 C1) and almost isointense on T2WI with irregular, blurred boundary (Figure 6 C2). DE-cMRI showed moderately enhanced signal on the scar tissue (Figure 6 C3). With impaired motion and thickening at the lateral and inferior wall, the lesion signal was partially lost on the fat suppressed cine-images, suggesting the initiation of LM.

LM after MI (Figure 6 D1-D5, E1, E2): 9 months after MI, T1WI exhibited hyperintense signal with clear boundary at the lateral wall (Figure 6 D1), along with a dramatic signal drop on *ex vivo* fat suppressed T1WI (Figure 6 E1). T2WI showed moderately hyperintense signal (Figure 6 D2) correspondent to the signal-void area on *ex vivo* fat suppressed T2WI (Figure 6 E2). DE-cMRI did not reveal apparent contrast enhancement of this healed MI (Figure 6 D3). On fat suppressed cine-images, the signal was lost at the lesion, of which the area was enlarged relative to that 5 months ago (Figure 6 D4 and D5 vs. C4 and C5), suggesting the formation of LM.

### Pathological findings after 9 months MI

Macroscopically (Figure 6 F1-F3), cardiac sections including the fresh section (Figure 6 F1) and the same section 15 min (Figure 6 F2) and 24 hours (Figure 6 F3) after TTC staining corresponded well with cMRI. The thickness of the lateral and inferior wall appeared almost the same as that of the normal ventricular wall. The MI territory looked whitish and was not stained by TTC, suggesting that the scar has been extensively substituted by fat tissue. Part of posterior papillary muscle was also replaced by adipose tissue.

Low-power H&E (Figure 6 G1) and MTC (Figure 6 G3) slides confirmed the above gross inspection, and demonstrated the clear, definable interface between normal myocardium and LM of the ventricular wall. High-power H&E (Figure 6 G2) and MTC (Figure 6 G4) photomicrographs revealed that high densities of the adipose cells were surrounded by few fibers, and macrophages infiltrated the interstitial space.

In this case, the final LM sizes were approximately 25.13%, 24.87%, and 24.14% of the ventricular volume as quantified by in vivo T1WI, ex vivo T1WI and TTC.

**Figure 7:**
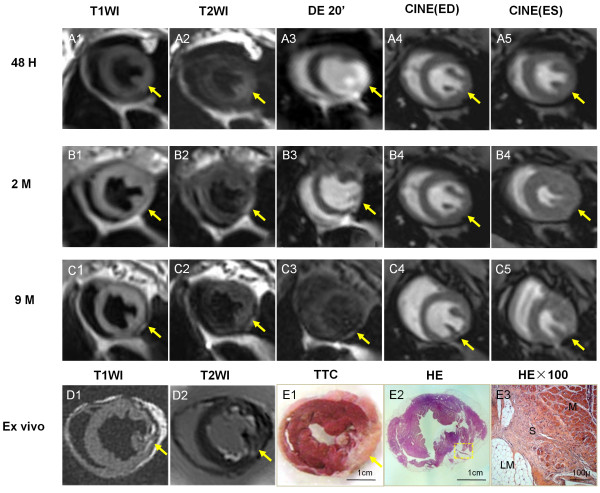
**Longitudinal evaluation of the changes of damaged myocardium (arrows), which progressively changed into scar tissue with moderate LM by in vivo and ex vivo cMRI in combination with postmortem histomorphology.** Serial images of the midcardiac slice by T1WI (A1-C1), T2WI (A2-C2), DE-MRI (A3-C3) and Cine-MRI (A4-C4; A5-C5), respectively, taken at 48 hours (A1-A5), 2 months (B1-B5) and 9 months (C1-C5). Acute (48 hours) MI (A1-A5): The infarct appeared as isointense region on T1WI (A1); more extensive hyperintense region on T2WI (A2); hyperenhanced area including papillary muscle at the lateral wall on DE-MRI (A3); and isointense but hypokinetic region at the lateral wall on Cine-MRI (A4, A5)**.** Chronic (2 months) MI (B1-B5): The lesion became almost isointense on both T1WI (B1) and T2WI (B2); moderately enhanced with blurred edge on DE-MRI (B3); and almost normal contraction and signals on the fat suppression Cine-MRI (B4, B5)**.** Healed (9 months) MI (C1-C5): The lesion appeared slightly hyperintense on T1WI (C1) and T2WI (C2); no contrast enhancement on DE-MRI (C3); and partially lost signals on the fat suppression Cine-images, suggesting occurrence of little LM (C4, C5). Ex vivo T1WI without fat suppression (D1): there exist patching high signals on the later wall. Ex vivo T2WI with fat suppress (D2): the signal void area corresponded to the region with high signal on T1 WI, indicating the region of little LM. Photograph of macroscopic TTC section (E1): the clear distinction between normal myocardium and TTC negative region is observed. Photograph of macroscopic HE stained slide (E2): the region corresponding to TTC negative area appeared inhomogeneous, suggesting mixed scar tissues. Dashed square indicates where microscopy was focused. Photomicrograph of HE staining (E3) proves the presence of the adipose cells mixed with fibrotic scar (magnification, ×100). N: normal myocytes; S: scar tissue; LM: lipomatous metaplasia.

The other 2 rabbits manifested smaller LM 9 months after MI as exemplified on Figure 7. Interestingly, although LM was hardly detectable by cMRI in this case (Figure 7 A-E), photomicrographs do demonstrate sporadic or patchy presence of adipose deposition in the lateral ventricular wall and posterior papillary muscle (Figure 7 E1-E3), suggesting LM change to a lesser extent.

After 9 months of MI, all cases failed to show typical delayed enhancement on DE-MRI (Figures 6 and 7), suggesting ceased tissue reactions in the healed MI.

## Discussion

With an increasing importance in cardiovascular medicine, cMRI has been widely recognized as an accurate and reliable means for assessing cardiac anatomy and function, and for identifying different infarct components such as edema, necrotic tissue, fibrotic scar, fat or calcium after MI [8,12-15]. The black-blood spin echo sequences with T1WI and T2WI, the bright-blood steady-state sequences on cine MRI, and the contrast-enhanced cMRI are most useful for determining the presence, extent, and underlying pathophysiological changes in acute, subacute and chronic MI.

In this study, by using a clinic 3.0 T MRI scanner, we imaged rabbit MI models on both acute and chronic phases. The excellent correspondence of cMRI and histopathology confirmed the useful model of reperfused MI in rabbits and its application in cMRI studies at a clinic 3.0 T magnet. We were able to follow up the MI by cMRI for 9 months. The T2WI showed well demarcated hyperintensity in the acute MI as a result of cytotoxic and vasogenic edema 24 hours after the insult. This hyperintense region was larger than the contrast-enhanced region on DE-cMRI. The latter is regarded as the irreversible core of necrosis. Edema is generally maximal at 48 to 72 hours beyond myocardial ischemia and resolved over 2 weeks, as revealed on T2WI [11,16]. The infarct did not appear much differently on T1WI between 24 hours and 2 weeks, but became hypointense on T1WI at 2 months and was moderately enhanced on DE-cMRI, suggesting the formation of fibrotic scar. The signal intensity of MI remained enhanceable from acute through subacute until chronic stage at 2 months, but became unenhanced on DE-cMRI at 9 months. The functional studies of cine-MRI confirmed the systolic dysfunction of the left ventricle, showing a marked thinning and hypokinetic movement of the lateral and inferior wall. At month 9, the bright signal intensity characteristic of LM was demonstrated on both T1WI and T2WI, suggesting locally enriched fatty tissue of high proton density. This hyperintensity was drastically reduced by using a fat suppression technique as seen on *ex vivo* cMRI, indicating the presence of adipose LM. The diagnosis of LM was finally established by using the gold-standard histological staining methods that revealed the underlying adipose component in infarcted territories.

Since fibrotic scar was almost completely substituted by adipose tissue, the infarct could no longer be enhanced on DE-cMRI, which is in line with what was found in a patient [17,18]. Using MR fat-water separation imaging to evaluate chronic MI, the fat deposition was predominately found at mid myocardia or mid pericardia [19]. Other CT studies suggest that fat deposition often affects the subendocardium [20-22]. Interestingly, it was shown in our animal study that little fibrotic tissue was left and the fat infiltrated entirely from endocardium to epicedium within the infarct over several months. Therefore, the location and extension of adipose deposition in LM vary depending likely on the exact situations of individual MI patients, without a common pattern. Clinically, transformation of a compact scar into compressible and "sliding' adipose tissue may worsen ventricular wall function, thus facilitating and/or aggravating aneurysm formation [9]. However, this concern was not evident in our rabbit experiment, probably due to the difference of induced MI in a healthy heart from rabbits versus spontaneous MI in atherosclerotic human patients.

Although the lineage of adipocytes still remains unclear, preadipocytes are undifferentiated fibroblasts that could be stimulated to form adipocytes [23,24], or pluripotent myofibroblasts may differentiate into adipocytes. Not only the etiology and pathophysiology but also clinical aspects of the LM deserve research attentions. It is unclear whether the LM affects long-term prognosis. Substitution of scar tissue by LM is often associated with severe heart failure and is more frequently and extensively seen in CIHD [9]. It has been suggested that therapeutic approaches (e.g. ACE inhibitors and statins) in the management of IHD may promote the development of adiposity within scar tissue [24]. If confirmed, this also raises the intriguing possibility to therapeutically modify the underlying processes to promote myocyte, rather than adipocyte, generation. Our rabbit model may also be used in research for this purpose.

By combining *in vivo* longitudinal cMRI with postmortem histological verification, we were able to experimentally describe, for the first time to our knowledge, the LM phenomena. We noticed 1) the infarcted ventricular wall did not become thinning at 9 months after MI as expected; 2) the LM region was stained whitish by TTC in contrast to brick-red normal myocardium, which was confused with acute MI and left a question about its histological nature; 3) H&E photomicrographs indicate that damaged myocardium is replaced by large quantity of adipocytes infiltrated with macrophages, i.e. the MI was entirely substituted by LM; and 4) MTC stained photomicrographs reveal few collagen fibers left.

Post-infarct LM is increasingly reported in clinical patients with very limited histological evidence. It usually occurs several years or over one decade after MI [8,17,22,25,26], much longer than the cases in this animal study. This should not be surprising, because 9 months in rabbits approximate 8 years in humans to form LM, considering their lifespans in proportion. Therefore, the faster evolution of MI in rabbits may provide a good opportunity to study the mechanisms of LM and to develop new therapeutic interventions.

The rabbit appears an ideal animal for cardiac research [1,3,10,27]. Comparing to large animals such as pigs, sheep, and dogs, the mid-size rabbit can be easily handled in addition to its inexpensiveness. Small animals such as rats and mice need dedicated higher field MRI facilities, which are not universally available. We believe multiparametric capabilities of cMRI at a standard clinical magnet in combination with a rabbit model make it an ideal modality to noninvasively identify cardiac pathologies including LM *in vivo*.

## Study limitations

The major weakness of this work is the small sample size and only 3 rabbits with chronic MI were studied longitudinally with cMRI, partially due to the constrains of animal protection and limited housing space. Secondly, this study was not by intention to address the LM, and therefore only some cMRI sequences were applied for detection of fat. The LM was only recognized incidentally at 9 months after MI induction. Nevertheless, the collected evidences especially from the evolving features on cMRI and the characteristic changes on pathological specimens do justify this reliable finding of the LM in rabbits.

## Conclusion

We validated 3.0 T cMRI for evaluation of acute and chronic MI in rabbits and presented a new finding of the LM in rabbits with chronic MI after a longitudinal (from 48 hours to 9 months) follow-up using cMRI in correlation with histopathology. The *in vivo* cMRI corresponded well to *ex vivo* MRI and histomorphology, suggesting a promising animal model and research platform for further study on the mechanisms and possible therapeutic interventions of LM.

## Competing interests

All authors claim no conflict of interest what so ever in the context.

## Authors’ contributions

All the authors 1) have made substantial contributions to conception and design, or acquisition of data, or analysis and interpretation of data; 2) have been involved in drafting the manuscript or revising it critically for important intellectual content; and 3) have given final approval of the version to be published.

## Pre-publication history

The pre-publication history for this paper can be accessed here:

http://www.biomedcentral.com/1471-2342/13/18/prepub

## References

[B1] Fonge H Vunckx K Wang H Feng Y Mortelmans L Nuyts J Non-invasive detection and quantification of acute myocardial infarction in rabbits using mono-[123I]iodohypericin microSPECT Eur Heart J 2008 29 2 260 269 18156139 10.1093/eurheartj/ehm588

[B2] Feng Y Xie Y Wang H Chen F Ye Y Jin L A modified rabbit model of reperfused myocardial infarction for cardiac MR imaging research Int J Cardiovasc Imaging 2009 25 3 289 298 10.1007/s10554-008-9393-2 19043805

[B3] Feng Y Xie Y Wang H Chen F Marchal G Ni Y Animal models of ischemic heart disease for cardiac MR imaging research International Journal of Modelling, Identification and Control 2010 9 288 310 10.1504/IJMIC.2010.032809

[B4] Greenman RL Shirosky JE Mulkern RV Rofsky NM Double inversion black-blood fast spin-echo imaging of the human heart: a comparison between 1.5T and 3.0T J Magn Reson Imaging 2003 17 6 648 655 10.1002/jmri.10316 12766893

[B5] Hinton DP Wald LL Pitts J Schmitt F Comparison of cardiac MRI on 1.5 and 3.0 Tesla clinical whole body systems Invest Radiol 2003 38 7 436 442 12821858 10.1097/01.RLI.0000067489.31556.70

[B6] Kimura F Matsuo Y Nakajima T Nishikawa T Kawamura S Sannohe S Myocardial fat at cardiac imaging: how can we differentiate pathologic from physiologic fatty infiltration? Radiographics 2010 30 1 1587 1602 21071377 10.1148/rg.306105519

[B7] van den Borne SWM Diez J Blankesteijn WM Verjans J Hofstra L Narula J Myocardial remodeling after infarction: the role of myofibroblasts Nat Rev Cardiol 2010 7 1 30 37 10.1038/nrcardio.2009.199 19949426

[B8] Lucke C Schindler K Lehmkuhl L Grothoff M Eitel I Schuler G Prevalence and functional impact of lipomatous metaplasia in scar tissue following myocardial infarction evaluated by MRI Eur Radiol 2010 20 9 2074 2083 10.1007/s00330-010-1791-x 20407897

[B9] Baroldi G Silver MD De Maria R Parodi O Pellegrini A Lipomatous metaplasia in left ventricular scar Can J Cardiol 1997 13 1 65 71 9039067

[B10] Agudelo CF Fictum P Skoric M Kazbundova K Svoboda M Scheer P Unusual massive fatty infiltration of the heart in a British cat: a case report Vet Med 2011 56 3 145 147

[B11] Choi SH Kang JW Kim ST Lee BH Chun EJ Schuleri KH Investigation of T2-weighted signal intensity of infarcted myocardium and its correlation with delayed enhancement magnetic resonance imaging in a porcine model with reperfused acute myocardial infarction Int J Cardiovasc Imaging 2009 25 Suppl 1 111 119 19184523 10.1007/s10554-009-9425-6

[B12] Saeed M Lee RJ Weber O Do L Martin A Ursell P Scarred myocardium imposes additional burden on remote viable myocardium despite a reduction in the extent of area with late contrast MR enhancement Eur Radiol 2006 16 4 827 836 10.1007/s00330-005-0052-x 16362420

[B13] Aquaro GD Nucifora G Pederzoli L Strata E De Marchi D Todiere G Fat in left ventricular myocardium assessed by steady-state free precession pulse sequences Int J Cardiovasc Imaging 2012 28 4 813 821 10.1007/s10554-011-9886-2 21562725

[B14] Okayama S Uemura S Sugimoto H Enomoto S Onoue K Omori S Dual gradient-echo in-phase and opposed-phase magnetic resonance imaging to evaluate lipomatous metaplasia in patients with old myocardial infarction Magn Reson Med Sci 2010 9 2 85 89 10.2463/mrms.9.85 20585199

[B15] Okayama S Uemura S Watanabe M Morikawa Y Onoue K Soeda T Novel application of black-blood echo-planar imaging to the assessment of myocardial infarction Heart Vessels 2010 25 2 104 112 10.1007/s00380-009-1172-z 20339971

[B16] Arai AE Magnetic resonance imaging for area at risk, myocardial infarction, and myocardial salvage J Cardiovasc Pharmacol Ther 2011 16 3–4 313 320 21821534 10.1177/1074248411412378 PMC8690274

[B17] Valle-Munoz A Estornell-Erill J Corbi-Pascual M Ridocci-Soriano F Lipomatous metaplasia. Two chronic infarcts in the same patient detected by cardiac magnetic resonance Rev Esp Cardiol 2009 62 7 830 831 10.1016/S0300-8932(09)71703-8 19709525

[B18] Arnold JR Karamitsos TD Pegg TJ Francis JM Neubauer S Left ventricular lipomatous metaplasia following myocardial infarction Int J Cardiol 2009 137 1 e11 e12 10.1016/j.ijcard.2008.05.039 18674834

[B19] Goldfarb JW Roth M Han J Myocardial fat deposition after left ventricular myocardial infarction: assessment by using MR water-fat separation imaging Radiology 2009 253 1 65 73 10.1148/radiol.2532082290 19703860

[B20] Wheeler LD Wood AM Re: CT imaging features and frequency of left ventricular myocardial fat in patients with CT findings of chronic left ventricular myocardial infarction Clin Radiol 2008 63 10 1184 1185 author reply 1185 18774369 10.1016/j.crad.2008.03.013

[B21] Zafar HM Litt HI Torigian DA CT imaging features and frequency of left ventricular myocardial fat in patients with CT findings of chronic left ventricular myocardial infarction Clin Radiol 2008 63 3 256 262 10.1016/j.crad.2007.08.007 18275865

[B22] Ichikawa Y Kitagawa K Chino S Ishida M Matsuoka K Tanigawa T Adipose tissue detected by multislice computed tomography in patients after myocardial infarction JACC Cardiovasc Imaging 2009 2 5 548 555 10.1016/j.jcmg.2009.01.010 19442939

[B23] Lehmann GM Woeller CF Pollock SJ O’Loughlin CW Gupta S Feldon SE Novel anti-adipogenic activity produced by human fibroblasts Am J Physiol Cell Physiol 2010 299 1 c672 c681 20554910 10.1152/ajpcell.00451.2009 PMC2944318

[B24] Su L Siegel JE Fishbein MC Adipose tissue in myocardial infarction Cardiovasc Pathol 2004 13 2 98 102 10.1016/S1054-8807(03)00134-0 15033159

[B25] Schmitt M Samani N McCann G Images in cardiovascular medicine. Lipomatous metaplasia in ischemic cardiomyopathy: a common but unappreciated entity Circulation 2007 116 1 e5 e6 10.1161/CIRCULATIONAHA.107.690800 17606850

[B26] McKeag NA Harbinson MT McKeown PP Roberts MJ Lipomatous metaplasia within an old anterior myocardial infarction Qjm-an International Journal of Medicine 2011 104 12 1101 1102 10.1093/qjmed/hcq216 21081563

[B27] van der Laarse A van der Wall EE Rabbit models: ideal for imaging purposes? Int J Cardiovasc Imaging 2009 25 3 299 301 10.1007/s10554-008-9401-6 19085084

